# The single-dose Janssen Ad26.COV2.S COVID-19 vaccine elicited robust and persistent anti-spike IgG antibody responses in a 12-month Ugandan cohort

**DOI:** 10.3389/fimmu.2024.1384668

**Published:** 2024-05-08

**Authors:** Jennifer Serwanga, Laban Kato, Gerald Kevin Oluka, Violet Ankunda, Jackson Sembera, Claire Baine, Isaac Kitabye, Angela Namuyanja, Solomon Opio, Joseph Ssebwana Katende, Peter Ejou, The COVID-19 Immunoprofiling Team, Pontiano Kaleebu

**Affiliations:** ^1^ Department of Immunology, Uganda Virus Research Institute, Entebbe, Uganda; ^2^ Viral Pathogens Research Theme, Medical Research Council, Uganda Virus Research Institute and London School of Hygiene and Tropical Medicine, Uganda Research Unit, Entebbe, Uganda

**Keywords:** Janssen Ad26.COV2.S vaccine, SARS-CoV-2 immunity, spike protein antibodies, nucleocapsid protein antibodies, Ugandan vaccine cohort, single-dose vaccination, breakthrough infections, antibody persistence

## Abstract

**Introduction:**

The study investigation examined the immune response to the Janssen Ad26.COV2.S COVID-19 vaccine within a Ugandan cohort, specifically targeting antibodies directed against spike (S) and nucleocapsid (N) proteins. We aimed to examine the durability and robustness of the induced antibody response while also assessing occurrences of breakthrough infections and previous anti-Spike seropositivity to SARS-CoV-2.

**Methods:**

The study included 319 specimens collected over 12 months from 60 vaccinees aged 18 to 64. Binding antibodies were quantified using a validated ELISA method to measure SARS-CoV-2-specific IgG, IgM, and IgA levels against the S and N proteins.

**Results:**

The results showed that baseline seropositivity for S-IgG was high at 67%, increasing to 98% by day 14 and consistently stayed above 95% for up to 12 months. However, S-IgM responses remained suboptimal. A raised S-IgA seropositivity rate was seen that doubled from 40% at baseline to 86% just two weeks following the initial vaccine dose, indicating sustained and robust peripheral immunity. An increase in N-IgG levels at nine months post-vaccination suggested breakthrough infections in eight cases. Baseline cross-reactivity influenced spike-directed antibody responses, with individuals harbouring S-IgG antibodies showing notably higher responses.

**Discussion:**

Robust and long lasting vaccine and infection-induced immune responses were observed, with significant implications for regions where administering subsequent doses poses logistical challenges.

## Introduction

The COVID-19 pandemic, brought about by the emergence of the novel SARS-CoV-2 virus, rapidly escalated into a global health emergency of unprecedented proportions. The advent of vaccines emerged as a beacon of hope (1), providing relief to countries like Uganda, already strained healthcare resources were further stretched by the profound impact of the pandemic. The Ad26.COV2.S vaccine by Johnson & Johnson–Janssen is a recombinant human adenovirus type 26 (Ad26) vector. It carries a full-length, prefusion-stabilized SARS-CoV-2 spike protein, encoding it within its membrane. The single-dose Janssen Ad26.COV2.S COVID-19 vaccine, strategically prioritized in Uganda for key demographic groups such as teachers and hard-to-reach populations, including mobile and remote communities, due to its logistical practicality, offered a valuable opportunity to examine the immune responses induced by this vaccine within a sub-Saharan African context. This study informs local public health strategies and contributes to the global discourse on vaccine efficacy.

Emerging research has underscored the critical role of spike (S)-directed (2, 3) immune responses in conferring protection against SARS-CoV-2 (4). Prevailing literature suggests that vaccine-induced immunity against SARS-CoV-2 can significantly vary across vaccine types and populations (5–8). While most studies have concentrated on spike (S) protein-directed immune responses (7, 9), few study has explored the concurrent context of nucleocapsid protein (N)-directed responses, especially in the setting of the spike protein-based Janssen Ad26.COV2.S COVID-19 vaccine (10). Here, we monitored both spike (S) and nucleocapsid (N) protein-directed antibody responses, recognizing that N-directed responses, which are unexpected in spike-focused vaccines, could signal post-vaccination infections. This dual-tracking approach provided critical insights into the real-world effectiveness of the vaccine, shedding light not only its ability to provoke an immune response but also on its potential to prevent subsequent infections.

We hypothesized that the single-dose Janssen Ad26.COV2.S COVID-19 vaccine would elicit a robust immune response, and examined this through longitudinal analysis of 319 specimens from 60 individuals over 12 months. In our study, we measured both Spike (S-IgG) and Nucleocapsid (N-IgG) antibody responses. This approach enabled us to comprehensively delineate the patterns of seroconversion, assess the longevity of immune protection, and track the incidence of breakthrough infections. The significance of this study was augmented by the evolving landscape of the SARS-CoV-2 virus, especially with the concurrent emergence of new variants (11) that continually challenged the efficacy of existing vaccines (12, 13). By delving into the immune responses elicited by the Janssen Ad26.COV2.S COVID-19 vaccine in Uganda, our research informs global insights into its immunogenicity within the African context but also sets a precedent for similar investigations in other areas where Janssen Ad26.COV2.S COVID-19 vaccine has been pivotal (14, 15). This insight is crucial for informing vaccine-related public health strategies and policies, especially in regions where logistical challenges make single-dose vaccine regimens a more feasible option.

## Materials and methods

### Study population

We analyzed 319 specimens collected over 12 months from 60 individuals who received a single dose of the Janssen Ad26.COV2.S COVID-19 vaccine. Participant demographic characteristics are summarized in **Table 1**. Blood samples were obtained at baseline, immediately prior to vaccination, and at 14 and 28 days after the initial dose. Follow-up samples were taken at 6, 9, and 12 months after the initial dose. Study samples were collected during the real-world deployment of COVID-19 vaccines in Africa, aligning with the national imperative to safeguard lives. The national initiative did not mandate prior testing for infection status; the emphasis was on widespread coverage as the primary goal. Consequently, this study aligns with the Ministry of Health’s protocol and lacks data regarding previous infections. Thus, baseline S-IgG seropositivity stands as our surrogate measure for estimating prior exposure. Samples were collected between November 15, 2021, and June 2, 2023, from vaccine-naïve individuals aged 18 to 64 years, with a median age of 22 years (IQR: 19-25 years), during the epidemiologic waves of SARS-CoV-2 variants outlined in **Supplementary Table 2**. The cohort comprised 13 females (21.7%) and 47 males (78.3%). Baseline blood samples were obtained from 58 of 60 participants. These individuals were subsequently classified based on their baseline S-IgG responses measured at day 0. Subjects with S-IgG levels above the established cut-off were classified as baseline S-IgG positive (S-IgG+), while those below this threshold were considered baseline S-IgG negative (S-IgG-). Among the 58 subjects, 39 (67%) were S-IgG +, contributing 218 samples, while 19 (33%) were S-IgG-, providing 95 samples. This categorization at baseline provided a foundational reference for analyzing S-IgG responses in our cohort. Reinfections are typically detected through genomic sequencing of nasopharyngeal swab samples (16). Various methods have been employed to differentiate between reinfection and initial infection. For instance, one macaque study suggested that a 7.6-fold increase in N-IgG antibody levels could indicate reinfection (14), while a study in West Africa proposed a 7-fold rise (15). Similar trends were observed in studies conducted in high-income settings (17, 18). Our previous serological analysis of two confirmed SARS-CoV-2 reinfection cases in this population, validated by rt-PCR, revealed an 11-fold surge in N-IgG antibody concentration following reinfection (3). To strengthen our conclusion of the absence of reinfection, we established a more stringent criterion, requiring no more than a 2-fold increase in N-IgG antibody concentration.

**Table 1:** Characteristics of the overall participants, n = 60.

Characteristic	N (%)
Total participants	60
**Age**, Median (IQR)	22 (19-25)
Gender	
Male	47 (78.3)
Females	13 (21.7)

### Binding antibody ELISA to detect SARS-CoV-2-specific IgG, IgM, and IgA levels

We used a validated ELISA (19, 20), to detect the presence of SARS-CoV-2-specific IgG, IgM, and IgA antibodies against the spike (S) and nucleocapsid (N) proteins. Both the Spike and nucleocapsid were recombinant proteins based on the ancestral SARS-CoV-2 (NCBI Accession numbers: YP_009724390.1 and YP_009724397.2). ELISA plates were coated with antigen at a concentration of 3 μg/ml, which had been verified to have the highest possible specificity and sensitivity. The OD values were measured at 450 nm to quantify antibody concentrations in nanograms per millilitre (ng/ml). Seropositivity was determined using previously established cut-off OD values specific to this population, as described before (20). The OD seropositivity thresholds were 0.432 for IgG, 0.459 for IgM, 0.226 for IgA for spike-specific antibodies, 0.454 for IgG, 0.229 for IgM, and 0.225 for IgA for nucleocapsid protein-specific antibodies. These values were determined from an extensive analysis of a large sample population.

### Statistical analysis

Seroconversion percentages at each follow-up time point were visualized using diverging bar graphs. Boxplots were used to compare medians (represented by horizontal lines), means (indicated by black dots), and quartile ranges (denoted by the top and bottom edges of the box). The Wilcoxon test, with Hochberg correction for multiple testing adjustments, was conducted to determine differences in antibody responses between pairwise comparisons at different time points. Unpaired tests were selected due to missing data at various time points, and a significance threshold of p > 0.05 indicated non-significance (ns). Statistical significance was denoted as follows: * for p ≤ 0.05, ** for p < 0.01, *** for p < 0.001, and **** for p < 0.0001.

## Results

### Dynamic patterns of seroconversion and long-lasting immunity post-vaccination with the Janssen Ad26.COV2.S COVID-19 vaccine

**Figure 1:**
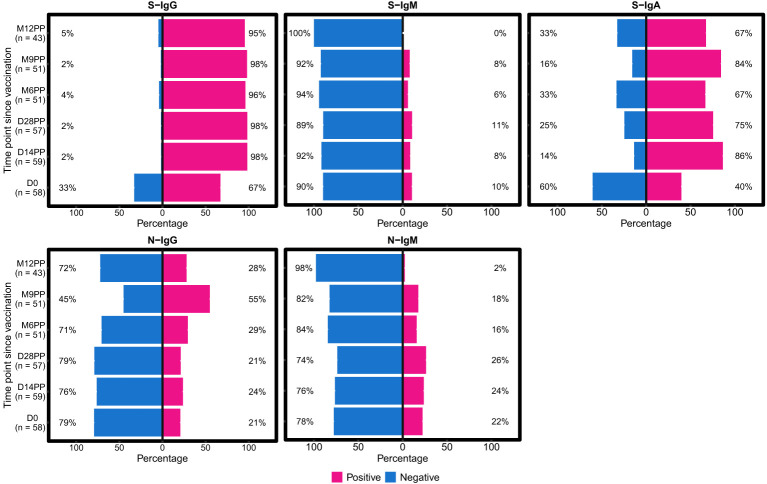
Twelve-Month Longitudinal Study of Seroconversion Dynamics Using S- and N-Protein-Directed Antibody Detection in Individuals Vaccinated with Janssen Ad26.COV2.S COVID-19 Vaccine. This figure displays the percentage of subjects seroconverting against S (spike) and N (nucleocapsid) proteins, segmented by the detection of S-IgG, S-IgM, S-IgA and N-IgG and N-IgM antibodies. Data is stratified based on baseline S-IgG seropositivity: baseline positives are indicated in pink, and negatives in blue. N-directed antibodies were monitored as a proxy for predicting potential infection, categorized as either IgG (indicating previous exposure) or IgM (indicating current exposure).

This study presents evidence of longitudinal seroconversion patterns in response to vaccination with the single dose Janssen Ad26.COV2.S COVID-19 vaccine in Uganda. The data, captured over 12 months since initial vaccination, show the temporal changes in various immunoglobulin responses following a priming dose of the vaccine, illustrated in **Figure 1**. The study demonstrated a marked and sustained increase in S-IgG positivity, from 67% at baseline to 98% by day 14 post-priming (D14PP). This elevated seropositivity persisted till 12-months, highlighting the vaccine’s ability to elicit a durable and robust hybrid immune response.

In contrast, initial S-IgM responses were minimal, with only 10% seropositivity at day 0, maintaining these levels over an extended period, until 12 months (M12PP) when the seropositivity rate eventually reducing to 0%. The decline in S-IgM seroprevalence highlights the typical switch to a predominantly IgG-mediated immune responses (21). Meanwhile, S-IgA seropositivity substantially rose from a 40% baseline seropositivity to 86% by day 14 post-prime (D14PP). This seropositivity was sustained over the 12-month follow-up, with proportions at 75%, 67%, and 84% at subsequent intervals, concluding with 67% at the 12-month mark. Approximately 70% of subjects exhibited consistent S-IgA seropositivity throughout the study following the first vaccine dose, indicating durable serum IgA titres. N-IgG responses were detected in 21% of subjects at baseline, with a notable rise to 55% nine months after the primary dose (M9PP), followed by a drop to 28% at 12 months. This indicates an increase in breakthrough infections between 6 months (M6PP) and 9 months (M9PP) after vaccination. N-IgM seropositivity exhibited an initial modest increase from a baseline of 22% to 26% by 28 days post-primary dose, followed by a decline to 16% at six months, and ultimately reached low level of 2% by 12 months post-vaccination. The persistent levels of S-IgG and S-IgA seropositivity, implies the vaccine’s effectiveness in inducing a robust and lasting immune response, but as these responses were more pronounced among individuals who were S-IgG seropositive at baseline, the role of prior infection or antigenic exposure is highlighted. These results show the longevity of Janssen Ad26.COV2.S COVID-19 vaccine-induced immunity in a context of prior infection/antigenic exposure, within the landscape of a continuing epidemic, which is crucial for evaluating the effectiveness of dosing schedules, shaping future vaccination strategies and informing public health policies.

### A temporal analysis of the evolving antibody response dynamics following vaccination

**Figure 2:**
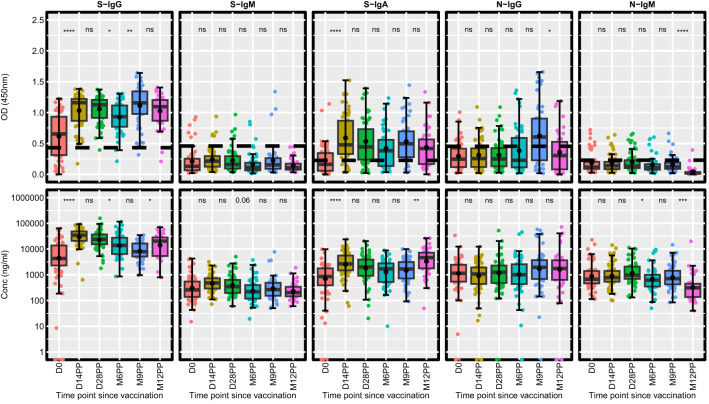
Longitudinal Analysis of Antibody Responses Over 12 months Following Administration of the Single-Dose Janssen Ad26.COV2.S COVID-19 Vaccine. This figure illustrates the antibody response levels, measured in optical density (OD) and concentration (ng/ml), throughout the study period. Each boxplot displays the interquartile range, with the mean represented by a black solid circle and the median by a horizontal line. Statistical analysis of the antibody response variation over time was conducted using an unpaired Wilcoxon test, with a Hochberg correction for multiple comparisons. Significance thresholds are indicated as: ‘ns’ for p > 0.05 (non-significant), ‘*’ for p ≤ 0.05, ‘**’ for p < 0.01, ‘***’ for p < 0.001, and ‘****’ for p < 0.0001.

Our analysis delineated the temporal dynamics of antibody responses post-vaccination (**Figure 2**). After conducting unpaired Wilcoxon tests with Hochberg corrections for multiple comparisons, we observed a notable increase in S-IgG OD values and antibody concentrations 14 days post-vaccination. The rise in S-IgG concentrations reached a plateau by day 28 post-vaccination (D28PP). However, a marked decline in these antibodies was observed by month six post-vaccination (M6PP), indicating a time-dependent waning of immunity (**Table 2**). A notable surge in S-IgG antibody levels detected at 12 months may indicate a significant increase in the number of breakthrough infections, as shown in **Table 1**. However, the surge in N-IgG between 6 and 9 months suggests that breakthrough infections were already occurring after 6 months post-vaccination. In contrast, S-IgM antibody OD levels and concentrations were largely suboptimal. The S-IgA antibodies showed an immediate significant post-vaccination increase, a gradual decline, and a notable resurgence 9 months after vaccination, possibly indicative of re-infection/breakthrough infection. Throughout the study, N-IgG responses period remained predominantly low failing to reach optimal thresholds, except for the marginal, non-significant increase observed at 9- months, as attributed to some breakthrough infections in this period. In parallel, N-IgM levels maintained a consistently low profile, ending in a significant decrease at 12 months. These findings show the diversity of antibody responses following Janssen Ad26.COV2.S COVID-19 vaccine vaccination advancing our understanding of post-vaccination immunological processes.

**Table 2:** Summary statistics of the Spike-directed antibody responses over time.

Time point	Antibody	Median OD (IQR), 450nm	Median Conc (IQR), ng/ml	Median Conc (IQR), BAU/ml
D0	S-IgG	0.655 (0.312, 0.932)	4335.35 (2301.58, 13802.23)	71.405 (26.142, 227.131)
	S-IgM	0.129 (0.066, 0.252)	257.450 (138.530, 545.890)	9.964 (5.576, 20.607)
	S-IgA	0.162 (0.057, 0.345)	852.300 (389.250, 1784.05)	162.626 (74.253, 340.452)
D14PP	S-IgG	1.161 (0.867, 1.220)	34865.4 (20867.85, 48259.10)	653.075 (390.914, 903.925)
	S-IgM	0.214 (0.124, 0.296)	480.7 (291.65, 729.15)	18.20151 (11.226, 27.369)
	S-IgA	0.478 (0.330, 0.867)	2654.1 (1488.6, 5552.8)	506.5017 (284.065, 1059.722)
D28PP	S-IgG	1.127 (0.915, 1.205)	23856.05 (15389.02, 37047.03)	446.8804 (288.302, 693.934)
	S-IgM	0.161 (0.094, 0.295)	347.3 (196.5, 631.8)	13.27945 (7.715, 23.777)
	S-IgA	0.445 (0.239, 0.733)	2011.9 (912.7, 3889.8)	383.9372 (174.154, 742.336)
M6PP	S-IgG	0.934 (0.772, 1.115)	13886.6 (6439.6, 27783.0)	260.163 (120.688, 520.428)
	S-IgM	0.110 (0.065, 0.188)	217.0 (119.00, 399.65)	8.472 (4.856, 15.211)
	S-IgA	0.381 (0.136, 0.560)	1706.4 (525.70, 2769.95)	325.6322 (100.294, 528.612)
M9PP	S-IgG	1.157 (0.977, 1.342)	7924.6 (5162.125, 16456.775)	148.500 (96.762, 308.299)
	S-IgM	0.154 (0.090, 0.262)	264.9 (153.20, 485.45)	10.239 (6.118, 18.377)
	S-IgA	0.494 (0.278, 0.696)	1685.3 (723.75, 3116.80)	321.605 (138.093, 594.808)
M12PP	S-IgG	1.096 (0.867, 1.205)	20332.8 (5368.387, 28429.275)	380.893 (100.625, 532.532)
	S-IgM	0.110 (0.076, 0.173)	203.2 (145.3, 340.4)	7.963 (5.826, 13.025)
	S-IgA	0.418 (0.167, 0.565)	4656.4 (1773.65, 7231.85)	888.643 (338.467, 1380.170)

### Antibody fold changes and breakthrough infections following SARS-CoV-2 vaccination

**Figure 3:**
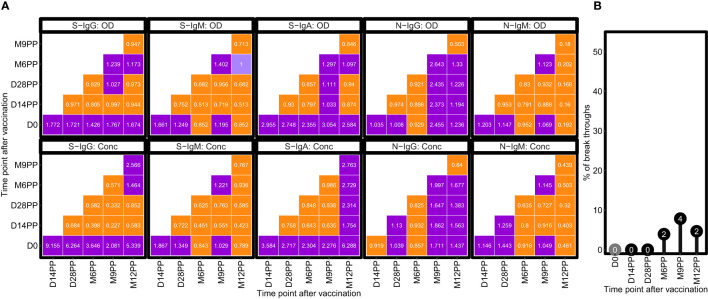
Temporal Dynamics of Median Antibody Response and Incidence of Breakthrough Cases Post-Vaccination. **(A)** illustrates the median fold changes in antibody responses between sequential time points. Fold changes are quantified as ratios, with a value of one indicating no change, values greater than 1 denoting an increase, and values less than one signifying a decrease. Increases in antibody responses are highlighted in red, decreases in green, and instances with no change are marked in orange. **(B)** delineates the prevalence of presumed infection and breakthrough cases in the study cohort, measured by the change in N-IgG antibody levels, before and after completion of the COVID-19 vaccination regimen. Grey circles indicate the percentage of subjects presumed infected at each time point before completing the vaccination regimen, while black circles represent the percentage of breakthrough cases post-full vaccination. The y-axis quantifies these percentages. Breakthrough cases, defined as subjects with an 11-fold increase in N-IgG levels indicative of infection occurring 14 days or more after the complete vaccination, amounted to three individuals, all of whom were identified six months post-vaccination.

Fold change analyses revealed notable increases in antibody responses following vaccination, with S-IgG and S-IgA OD levels exhibiting 2-fold and 3-fold elevations respectively, within 14 days after the primary vaccination (**Figure 3**). This initial surge reached a stable plateau, as evidenced by negligible fluctuations in OD levels at subsequent time intervals. A modest 1.2-fold rise in S-IgM OD levels occurred two weeks post-prime, alongside minimal changes in N-IgG and N-IgM, reflecting a comparatively subdued N-directed response. More pronounced changes were observed in antibody concentrations, with S-IgG concentrations surged, registering over 9-fold and 6-fold increases at 14- and 28-days post-prime, respectively. S-IgA concentrations also rose significantly, showing 3.5-fold and 3-fold increments at the same time points. However, N-directed IgG and IgM concentrations remained relatively unchanged throughout the study, as summarized in **Figure 3A**. Subjects were categorized as breakthrough cases if they demonstrated an 11-fold or greater rise in N-IgG concentration, as described before (3), indicative of infection, occurring at least 14 days after completion of the vaccination schedule. A total of eight breakthrough COVID-19 cases, occurring at six, nine, and twelve months post-primary vaccination with the Janssen Ad26.COV2.S COVID-19 vaccine, were identified through analysis. Of the total breakthrough cases observed, three occurred in individuals initially negative for baseline S-IgG, while five cases manifested in those initially positive, as outlined in **Figure 3B**.

### Impact of baseline cross-reactivity on spike-directed antibody responses post-vaccination

**Figure 4:**
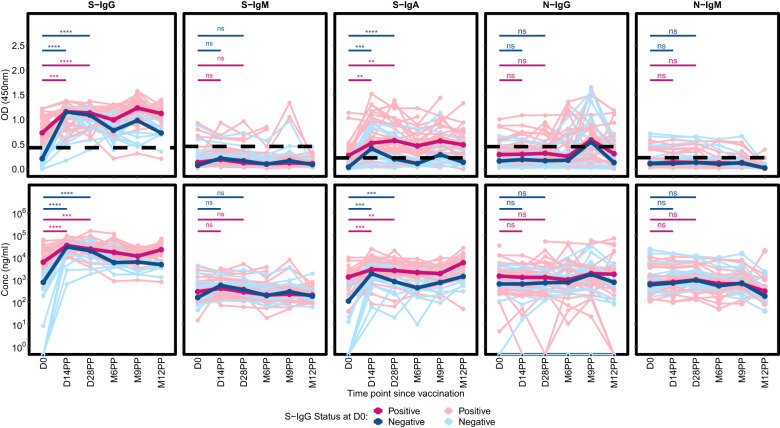
Comparative Profiling of Median Spike-Directed Antibody Responses Post-Janssen Ad26.COV2.S COVID-19 Vaccination Stratified by Baseline S-IgG Seropositivity. This figure depicts the individual trends in Spike-directed antibody responses (light-shaded lines) and the median responses (dark, thicker lines), categorized based on baseline S-IgG antibody levels. Subjects are classified as S-IgG positive (shown in red) if their baseline S-IgG levels are at or above the established cutoff value and S-IgG negative (illustrated in blue) if below this threshold. The data tracks these antibody responses over 12 months following the initial vaccination, providing a detailed temporal view of the immune response elicited by the Ad26.COV2.S COVID-19 Janssen vaccine. Differences in antibody responses between D0 and D14PP as well as D0 and D28PP for each subgroup were assessed using a Wilcoxon test. Significance bars (red for baseline S-IgG positive and blue for baseline S-IgG negative) indicate the levels of significance;: ‘ns’ for p > 0.05 (non-significant), ‘*’ for p ≤ 0.05, ‘**’ for p < 0.01, ‘***’ for p < 0.001, and ‘****’ for p < 0.0001.

Distinct patterns in Spike-directed antibody responses were identified based on S-IgG serostatus at baseline. Participants with S-IgG antibody levels at or above the baseline cutoff were categorized as S-IgG positive (S-IgG+), while those below the threshold were labelled as S-IgG negative (S-IgG-). Significant increases in S-IgG responses were observed from baseline to both day 14 and day 28 post-prime in both groups, as confirmed by unpaired Wilcoxon tests, depicted in **Figure 4**. During the interval between Day 14 and Day 28 following initial vaccination, both S-IgG positive and negative cohorts exhibited a consistent plateau in optical density (OD) levels and concentrations of S-IgG antibodies, suggesting a critical window of immune response modulation during this timeframe, regardless of baseline serostatus. Following Day 28, a marked decrease in S-IgG levels was observed in the baseline S-IgG negative cohort, persistently remaining below those of the baseline positive cohort throughout the study, indicating the superiority of the antibody response elicited by multiple antigenic exposure among individuals who had prior infection. S-IgM antibody responses were consistently low in both groups, with most participants showing OD levels below the threshold throughout the follow-up period. Distinct disparities in S-IgA antibody responses were evident between individuals possessing pre-existing S-IgG (S-IgG+) and those lacking it (S-IgG-). Initially, baseline S-IgG+ subjects displayed S-IgA levels surpassing the established threshold, while S-IgG- counterparts exhibited lower S-IgA levels. Following primary vaccination, both groups demonstrated a significant rise in S-IgA levels by days 14 and 28 compared to baseline, with the S-IgG+ cohort consistently maintaining higher S-IgA responses than the S-IgG- group throughout the study. Both the S-IgG+ and S-IgG- cohorts consistently exhibited low median levels of N-IgG and N-IgM antibodies, with marginal disparities between them. A slight elevation in N-IgG concentrations was detected between 6 and 9 months, followed by a subsequent decline below the predefined threshold. These observations are further substantiated upon exclusion of participants that had subsequent infections or re-infections (**Supplementary Figure 1**) shown by a substantial 11-fold increase in N-IgG.

## Discussion

In this study, we assessed the immune response elicited by the single-dose Janssen Ad26.COV2.S COVID-19 vaccine within a Ugandan cohort, strategically prioritized for selected key demographic populations, such as teachers, highly mobile populations, and residents in remote hard-to-reach areas. This approach provided crucial data on the vaccine’s effectiveness in diverse, and in logistically challenging communities, often underserved in healthcare. Our investigation revealed a significant and enduring rise in S-IgG responses post-administration of the Janssen Ad26.COV2.S COVID-19 vaccine. Within 14 days of the initial dose, S-IgG seropositivity surged from 67% at baseline to 98%, sustaining these heightened levels throughout the observed duration. This result corroborates previous studies demonstrating the vaccine’s effectiveness in generating robust and long-lasting spike-specific antibodies (8, 22, 23). This is particularly significant in scenarios where administering subsequent doses poses logistical hurdles. However, we observed higher concentrations of S-IgG and S-IgA, as well as more durable S-IgA in subjects that had previous antigen exposure through prior-infection, suggesting an advantage of multiple vaccine-doses analogous to the multiple vaccine-exposure, unlike the single-dose regimen for the Ad26.COV2.S vaccine used in this study.

We observed a significant increase in nucleocapsid protein-directed IgG (N-IgG) and IgM (N-IgM) antibodies in eight fully vaccinated recipients of the Janssen Ad26.COV2.S COVID-19 vaccine. This surge in N-IgG and N-IgM levels, contrary to the vaccine’s target on the spike protein, implies potential breakthrough infections (3). These findings inform post-vaccination infection rates and underscore the importance of continuous serological surveillance to guide booster (24)vaccinations. The observed 13% breakthrough rate (8 out of 60) post-Janssen vaccine administration closely mirrors the 10% rate (6 out of 60) reported in comparative studies using the Coronavac COVID-19 vaccine (Sinovac) within the same demographic cohort during the concurrent period (1). Recent studies have demonstrated comparable trends for Pfizer-BioNTech’s BNT162b2 and Moderna’s mRNA-1273 vaccines, with breakthrough rates of 23% (11 of 48) and 16% (3 of 19), respectively (24, 25). However, these comparisons should be interpreted cautiously due to the small sample sizes involved. The S-IgM responses were minimal, decreasing to zero by 12 months post-prime, aligning with the expected serological progression towards an IgG-dominant response (26–28), which was complemented by a marked increase in S-IgA responses post-vaccination with the Janssen Ad26.COV2.S COVID-19 vaccine. This pattern of prolonged immunity is consistent with the natural infection responses previously observed within this population (3). This study corroborates earlier South African research demonstrating sustained, robust spike-specific immune responses for up to six months post-administration of the Ad26.COV2.S vaccine, independent of prior infection history (29). However, in this study, the role of previous infection in augmenting the levels of S-IgG and S-IgA, as well as the durability of S-IgA was highlighted, as these parameters were better among subjects that were S-IgG seropositive at baseline. Furthermore, breakthrough infections were more frequent among the participants who were S-IgG seronegative at baseline, thus suggesting an advantage of multiple antigenic exposure in eliciting protective vaccine-induced antibodies. Our findings also align with responses elicited by other COVID-19 vaccines used in this demographic, such as Sinovac Biotech’s CoronaVac COVID-19 vaccine (30), the Oxford/AstraZeneca ChadOx1-S COVID-19 vaccine (31), the Pfizer-BioNTech BNT162b2 Vaccine (24), and Moderna’s mRNA 1273 (25) collectively supporting the vaccine’s effectiveness in this landscape and could have implications for future vaccination and public health strategies (32–34).

Our study demonstrates the elicitation of sustained immune responses to the Janssen Ad26.COV2.S COVID-19 vaccine, offering valuable insights for vaccine strategies in similar settings, complementing global data that often overlook regional variations in immune response due to demographic, genetic, and epidemiological factors (35, 36). The findings highlight the persistence of antibody responses for up to a year, despite observed breakthrough infections primarily occurring after six months, thus contributing to a broader understanding of vaccine-induced immunity against SARS-CoV-2.

Our methodology, though robust, needed to be improved by tracking breakthrough infections. The reliance on N-IgG as a post-vaccination infection marker may not fully represent the immune response spectrum of reinfections, particularly in cases with subdued secondary responses after boosting (37). Future studies should integrate viral sequencing and epidemiological insights to determine breakthrough infections and vaccine efficacy against diverse strains more accurately. The study’s analysis of antibody responses, while informative, could have been enriched by incorporating responses to other variants beyond the ancestral spike and nucleocapsid proteins, cellular immunity assessments for a fuller evaluation of vaccine efficacy and virus neutralization function studies. Constraints such as high baseline exposure and missing data at various points, necessitating the use of unpaired tests, may have impacted the robustness of our findings. Additionally, the unique demographic and epidemiological context of Uganda’s equatorial positioning suggests the need for further studies in diverse settings to enhance the generalizability of our results.

In conclusion, the single-dose Janssen Ad26.COV2.S COVID-19 vaccine demonstrated potent and lasting immune responses, which is crucial for remote and hard-to-reach populations. The rise in N-directed antibodies post-vaccination indicates possible breakthrough infections, underscoring the need for vigilant surveillance and adaptive vaccination strategies. These results contribute significantly to the global understanding of COVID-19 vaccine effectiveness, informing public health policy and vaccination strategies.

## Data availability statement

The original contributions presented in the study are included in the article/**Supplementary Material**. Further inquiries can be directed to the corresponding author.

## Ethics statement

The studies involving humans were approved by Research and Ethics Committee (GC/127/833) of the Uganda Virus Research Institute and the Uganda National Council for Science and Technology (HS637ES). The studies were conducted in accordance with the local legislation and institutional requirements. The participants provided their written informed consent to participate in this study. Written informed consent was obtained from the individual(s) for the publication of any potentially identifiable images or data included in this article.

## Author contributions

JS: Conceptualization, Data curation, Formal Analysis, Funding acquisition, Investigation, Methodology, Project administration, Resources, Supervision, Validation, Writing – original draft, Writing – review & editing, Visualization. LK: Data curation, Investigation, Validation, Writing – review & editing. GO: Investigation, Methodology, Validation, Writing – review & editing, Data curation, Writing – original draft. VA: Data curation, Formal Analysis, Visualization, Writing – original draft, Validation. JS: Data curation, Investigation, Writing – review & editing, Methodology, Validation. CB: Data curation, Investigation, Methodology, Validation, Writing – review & editing. IK: Data curation, Investigation, Writing – review & editing, Conceptualization. AN: Data curation, Investigation, Methodology, Validation, Writing – original draft. SO: Data curation, Investigation, Methodology, Validation, Writing – review & editing. JK: Data curation, Investigation, Methodology, Validation, Writing – review & editing. PE: Data curation, Investigation, Methodology, Validation, Writing – review & editing. T: Data curation, Investigation, Validation, Writing – review & editing. PK: Conceptualization, Formal Analysis, Project administration, Supervision, Writing – original draft, Writing – review & editing.

## Group member of the COVID-19 immunoprofiling team

Arthur Watelo Kalyebi^1^, Ivan Ssali^1^, Ben Gombe^1^, Susan Mugaba^1^ Hellen Nantambi^2^ Geoffrey Odoch^1^.

## Supplementary material

Supplementary Figure 1 Comparative Profiling of Median Spike-Directed Antibody Responses Post-Janssen Janssen Ad26.COV2.S COVID-19 Vaccination Stratified by Baseline S-IgG Seropositivity, excluding all subjects with breakthrough infection. **Supplementary Figure 1** shows Individual profiles of subjects over time categorized by S-IgG baseline seropositivity, excluding break through subjects. Total participants, n = 50, Baseline S-IgG+, n = 34 and baseline S-IgG-, n = 16. Subjects are classified as S-IgG positive (shown in red) if their baseline S-IgG levels are at or above the established cutoff value and S-IgG negative (illustrated in blue) if below this threshold. Differences in antibody responses between D0 and D14PP as well as D0 and D28PP for each subgroup were assessed using a Wilcoxon test. Significance bars (red for baseline S-IgG positive and blue for baseline S-IgG negative) indicate the levels of significance;: ‘ns’ for p > 0.05 (non-significant), ‘*’ for p ≤ 0.05, ‘**’ for p < 0.01, ‘***’ for p < 0.001, and ‘****’ for p < 0.0001.

Supplementary Table 1 Summary of predominant strains of SARS-CoV-2 during the study period.
