# Supplementary material for: The single-dose Janssen Ad26.COV2.S COVID-19 vaccine elicited robust and persistent anti-spike IgG antibody responses in a 12-month Ugandan cohort
Source: Front Immunol. 2024 May 8;15:1384668. doi: 10.3389/fimmu.2024.1384668 (PMC11109398; doi:10.3389/fimmu.2024.1384668)
Supplement: Supplementary Figure 1 — Comparative Profiling of Median Spike-Directed Antibody Responses Post-Janssen Janssen Ad26.COV2.S COVID-19 Vaccination Stratified by Baseline S-IgG Seropositivity, excluding all subjects with breakthrough infection. Supplementary Figure 1 shows Individual profiles of subjects over time categorized by S-IgG baseline seropositivity, excluding break through subjects. Total participants, n = 50, Baseline S-IgG+, n = 34 and baseline S-IgG-, n = 16. Subjects are classified as S-IgG positive (shown in red) if their baseline S-IgG levels are at or above the established cutoff value and S-IgG negative (illustrated in blue) if below this threshold. Differences in antibody responses between D0 and D14PP as well as D0 and D28PP for each subgroup were assessed using a Wilcoxon test. Significance bars (red for baseline S-IgG positive and blue for baseline S-IgG negative) indicate the levels of significance;: ‘ns’ for p > 0.05 (non-significant), ‘*’ for p ≤ 0.05, ‘**’ for p < 0.01, ‘***’ for p < 0.001, and ‘****’ for p < 0.0001. [file Presentation_1.pdf]

Supplementary Figures

Supplementary Figure 1: Comparative Profiling of Median Spike-Directed Antibody Responses Post-Janssen Janssen Ad26.COV2.S COVID-19 Vaccination Stratified by Baseline S-IgG Seropositivity, excluding all subjects with breakthrough infection

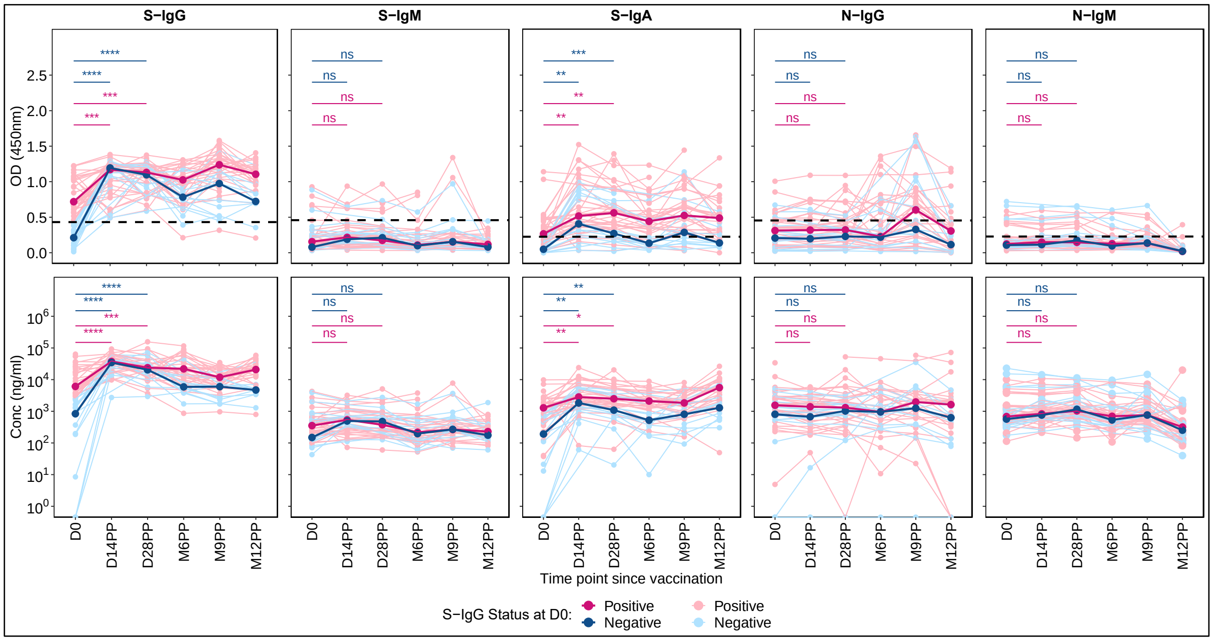

Supplementary Figure 1: Comparative Profiling of Median Spike-Directed Antibody Responses Post-Janssen Janssen Ad26.COV2.S COVID-19 Vaccination Stratified by Baseline S-IgG Seropositivity, excluding all subjects with breakthrough infection

Supplementary Figure 1 shows Individual profiles of subjects over time categorised by S-IgG baseline seropositivity, excluding break through subjects. Total participants, n = 50, Baseline S-IgG+, n = 34 and baseline S-IgG-, n = 16. Subjects are classified as S-IgG positive (shown in red) if their baseline S-IgG levels are at or above the established cutoff value and S-IgG negative (illustrated in blue) if below this threshold. Differences in antibody responses between D0 and D14PP as well as D0 and D28PP for each subgroup were assessed using a Wilcoxon test. Significance bars (red for baseline S-IgG positive and blue for baseline S-IgG negative) indicate the levels of significance; : 'ns' for $p > 0.05$ (non-significant), '*' for $p \leq 0.05$, '**' for $p < 0.01$, '***' for $p < 0.001$, and '****' for $p < 0.0001$.
