# Supplementary material for: The single-dose Janssen Ad26.COV2.S COVID-19 vaccine elicited robust and persistent anti-spike IgG antibody responses in a 12-month Ugandan cohort
Source: Front Immunol. 2024 May 8;15:1384668. doi: 10.3389/fimmu.2024.1384668 (PMC11109398; doi:10.3389/fimmu.2024.1384668)
Supplement: Supplementary Table 1 — Summary of predominant strains of SARS-CoV-2 during the study period. [file DataSheet_1.pdf]

Supplementary Tables

Supplementary Table 1: Summary of predominant strains of SARS-CoV-2 during the study period.

**Period**	**Predominant Strains (Waves of Variants)**
August 2020-February 2021	A23.1
May 2021-September 2021	B.1.617.2 (Delta)
December 2021	Emergence of Omicron (BA.1 and BA.1.1)
January 2022-March 2022	BA.1 and BA.1.1
April 2022-June 2022	BA.2, BA.2.31, BA.4, BA.4.1 and BA.5.2.1
July 2022-September 2022	BA.4.1, BA.5.2.1, BQ.1 and XBB.1
October 2022-January 2023	XBB.1, BQ.1 and XBB.3.2
